# Iodine-doped TiO_2_ nanotube coatings: a technique for enhancing the antimicrobial properties of titanium surfaces against *Staphylococcus aureus*

**DOI:** 10.1186/s13018-023-04354-8

**Published:** 2023-11-10

**Authors:** Xiu Yang, Neng-Fu Chen, Xiao-Li Huang, Shun Lin, Qing-Quan Chen, Wan-Ming Wang, Jin-Shui Chen

**Affiliations:** 1 Fuzong Clinical Medical College of Fujian Medical University, Fuzhou, 350000 China; 2 The 900th Hospital of Joint Logistic Support Force, PLA, Fuzhou, 350000 China; 3 Fuzhou Second Hospital, Fuzhou, 350000 China; 4 Fujian Medical University, Fuzhou, 350000 China

**Keywords:** Antibacterial coating, Titanium plate, Iodine, TiO_2_ nanotubes

## Abstract

**Background:**

Implant-related infections are a challenging complication of orthopedic surgery, primarily due to the formation of bacterial biofilms on the implant surface. An antibacterial coating for titanium implants was developed to provide novel insights into the prevention and treatment of implant-related infections.

**Methods:**

Titanium plates were coated with TiO_2_ nanotubes by anodization, and iodine was doped onto the coating via electrophoretic deposition. The obtained plates were characterized using a range of analytical techniques. Subsequently, *Staphylococcus aureus* was inoculated onto the surfaces of untreated titanium plates (control group), TiO_2_-nanocoated titanium plates (TiO_2_ group), and iodine-doped TiO_2_-nanocoated titanium plates (I-TiO_2_ group) to compare their antibacterial properties.

**Results:**

Twenty-four hour in vitro antimicrobial activity test of the I-TiO_2_ group against *Staphylococcus aureus* was superior to those of the other groups, and this difference was statistically significant (*P* < 0.05).

**Conclusions:**

This coating technology provides a new theoretical basis for the development of anti-infective implants against *Staphylococcus aureus* in orthopedics.

## Background

Infection is a prevalent and formidable complication of orthopedic implant placement and poses significant challenges to surgical practice. Despite the implementation of stringent anti-infection measures, various studies have revealed that the infection rates after total hip arthroplasty and total knee arthroplasty remain at 2.2 and 0.54% [1, 2]. Alarmingly, there are reports suggesting that pinhole infection rates associated with external fixation can reach as high as 70% [3]. Once an implant-related infection occurs, it necessitates a costly and time-consuming treatment, inflicting substantial physical and psychological distress on the patient. These infections impose an immense burden on the patients, their families, and national healthcare resources, especially when implant removal is also required. The primary etiology of medical implant-associated infections lies in the adhesion of bacteria to an implant surface, which subsequently leads to progressive bacterial biofilm (BBF) formation. In this context, Costerton et al. [4] initially proposed the biofilm theory while conducting research into its occurrence and development processes, i.e., attachment and adhesion, colonization, aggregation, maturation, and detachment. In addition, Gristina et al. [5] explored the relationship between bacterial biofilms and their hosts by introducing the concept of the “surface competition theory.” According to this theory, if host functional cells adhere to the surface of the prosthesis at the same time as the bacteria, the host cells will effectively “occupy” it; however, if bacteria dominate, prosthetic failure ensues. Furthermore, osseointegration can occur when the host cells prevail over the adherence of bacteria to the surfaces of prostheses. Upon initial adherence to an implant surface, the bacteria gradually develop into biofilms capable of resisting the immune attacks led by host cells through the production of extracellular matrix polymers [6].

To prevent bacterial adhesion and colonization, the modification and surface engineering of implants are current prominent areas of research. Various biocompatible materials that exhibit antibacterial properties have been investigated for the coating of endophyte surfaces. Common coatings employed to date include inorganic, organic, and anti-adhesive coatings, among others [7]. Inorganic antibacterial coatings based on metal ions (e.g., copper, silver, mercury, zinc, or palladium) or metal oxides (e.g., zinc oxide, titanium dioxide, magnesium oxide, or copper oxide) are commonly used. Although silver ion-based antimicrobial agents have received extensive attention because of their strong broad-spectrum antibacterial properties [8], biocompatibility issues and the potential cytotoxic effects associated with the release of silver ions cannot be overlooked [9]. Organic coatings are another major class of surface modification materials. These bioactive coatings transform implants into modules of sustained antibiotic release by incorporating antibiotics either inside or on the surface of the implant via direct or indirect means. Such a localized antibiotic delivery approach specifically targets infections [10]. Thus, by reducing the systemic toxicity and allowing precise control over drug release rates throughout the treatment duration, this method ensures consistent therapeutic dosing over an extended period without peaks or troughs. In addition, the ability to regulate the antibiotic release rate ensures a consistent therapeutic dosage, prevents fluctuations, and enables safe and prolonged drug release. However, Schierholz et al. [11] highlighted that an optimal local effect can only be achieved when the local antibiotic concentration is 1000 times higher than that in local lesions treated with intravenous antibiotics. Furthermore, controlling the sustained-release rate of drugs is challenging because short-term and high-concentration release can lead to tissue damage around the implant. Additionally, it remains uncertain whether loaded antibiotics are effective against pathogenic bacteria. Thus, the development and application of antibiotic coatings must be examined in much greater detail to address such issues.

As an alternative approach, adhesion barrier coatings aim to modify certain physical properties of the implant surface to prevent bacterial adhesion and colonization, with example target properties including the surface roughness, electrical energy, potential viscosity, and electrical conductivity [12, 13]. However, most antibacterial modifications for implants exhibit varying degrees of drawbacks, including an uncontrolled antibiotic release efficiency, histocompatibility, cytotoxicity concerns related to silver ion usage, and an inadequate antibacterial performance in the context of adhesion-blocking coatings. Consequently, no specific methods or materials have been identified for such applications.

The anodic oxidation method was previously employed by adding fluorine onto the surface of a titanium alloy to inhibit bacterial adhesion and combat biofilm formation [15, 16]. Consequently, iodine, another halogen, has also been investigated in the same context. Polyvinyl pyrrolidone-iodine (PVP-I) possesses a broad-spectrum antibacterial activity, a high efficacy, rapid bactericidal effects, a low drug toxicity, minimal tissue irritation, an excellent tissue permeability, and a prolonged duration of action. As a result, it has found extensive applications in clinical and nursing fields [14]. In addition, Shirai et al. [17] demonstrated the antibacterial properties and biocompatibility of iodine-coated implants. Therefore, iodine could potentially be surface-coated onto orthopedic titanium implants to impede bacterial adhesion.

Here, anodic oxidation is employed to modify the surface of orthopedic titanium plates, followed by the electrophoretic deposition of iodine onto the modified surface. This approach aims to develop an orthopedic titanium implant with inherent antibacterial properties while also providing a novel strategy for preventing and treating the infections associated with orthopedic implants.

## Methods

### Modification of orthopedic titanium plates using TiO_2_ nanotube arrays by anodic oxidation

Initially, an orthopedic titanium plate (3.0 cm length, 1.0 cm width, 0.3 cm thickness, Ti–6Al–4V, Dabo Medical, China) was polished using metallographic sandpaper specimens with gradually decreasing coarseness values (from P1000 to P4000) until no visible scratches remained on the sample surface. Subsequently, the plate was cleaned thoroughly under running water, followed by treatment with absolute ethanol to remove any oil residues or pickling effects. The surface was then etched using a mixture of hydrofluoric and nitric acids (1:3 ratio) for 30 s to enhance the roughness. Subsequently, ultrasonic cleaning was conducted sequentially using absolute ethanol, acetone, and double-distilled water for 20 min each, prior to drying the plate for subsequent use. To prepare the electrolyte solution required for anodization, deionized water (25 mL) was added to a glycerol solution (250 mL), followed by the addition of ammonium fluoride crystals (1.25 g). After vigorous mixing, a 0.5 wt.% NH_4_F glycerol electrolyte solution was obtained. During anodization, a magnet was placed inside the beaker containing the electrolyte solution for magnetic agitation. The pretreated titanium plate served as the anode and was connected to a power source (WYK-6005K DC power supply, DCSOON, Shenzhen, China) via an electrode line. A platinum plate (3.0 cm length, 1.0 cm width, 0.1 cm thickness) acted as the cathode and was connected via another electrode line. The electrodes were positioned parallel to one another and 2 cm apart. The temperature was set at 25 ℃, the stirrer speed was 130 rpm, the applied voltage was fixed at 70 V, and the duration of the anodization process was 10 h. Upon complete anodization, the samples were rinsed with deionized water, subjected to sonication for 10 min, and thoroughly dried prior to further use.

### Growth of the TiO_2_ nanotubes on the titanium plate surface

The growth parameters of the titanium dioxide nanotubes on the surfaces of the titanium plates were observed by electron microscopy.

### Electrodeposition on the Ti plate for iodine doping

**Fig. 1:**
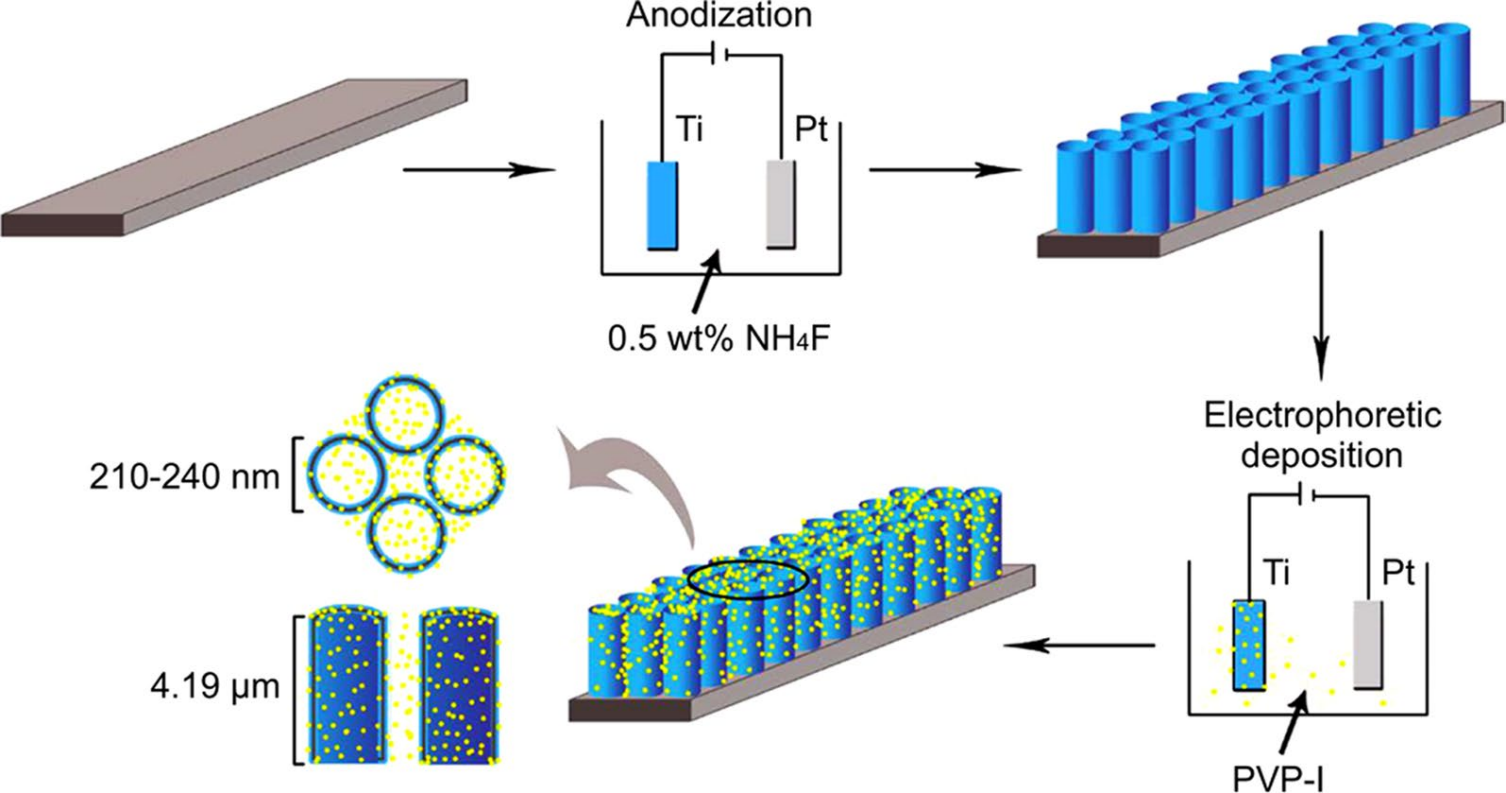
Technological pathway. Preparation of the iodine-doped TiO_2_ nanotube-coated titanium plates

A 4,000-ppm aqueous solution of PVP-I (1 ppm = 1 mg/kg = 1 mg/L) was prepared using distilled water (100 mL) and solid PVP-I powder (400 mg). The cathode consisted of a platinum plate (3 cm length, 1 cm width, 0.1 cm thickness), while the anode consisted of a TiO_2_ nanotube-deposited titanium plate, which was prepared as described above. After applying a voltage of 200 V for 30 min using a DC power supply (WYK-6005K DC power supply, DCSOON, Shenzhen, China), the titanium plate was coated with iodine-containing TiO_2_ nanotubes and cleaned with distilled water for 10 min prior to drying to obtain the desired iodine-doped TiO_2_ nanotube-coated titanium plate. The titanium plate was characterized using scanning electron microscopy (SEM), and the surface iodine content was determined using X-ray fluorescence spectrometry (Philips Company, Netherlands) and energy-dispersive spectrometer (Philips Company, Netherlands). Figure 1 illustrates the surface preparation of the titanium plate.

### In vitro antibacterial evaluation

Ten untreated titanium plates were used as control samples (Ti group), 10 TiO_2_ nanotube-coated titanium plates were classified as the TiO_2_ group, and 10 iodine-doped TiO_2_ nanotube-coated Ti plates were classified as iodine-doped TiO_2_ nanotube coating group (I-TIO_2_ group). All titanium plates were sterilized by fumigation with ethylene oxide. The bacterial suspension was prepared using a standard *Staphylococcus aureus* strain (ATCC25923) for which a turbidimeter with a scale of 0.5 was used to 1 mL of 1.5 × 10^8^ CFU/mL bacterial suspension. Subsequently, an aliquot (1 mL) of this suspension was diluted with sterilized tryptic soy broth to give a concentration of 1.5 × 10^6^ CFU/mL. Bacterial inoculation was then carried out by adding the prepared bacterial suspension (30 mL) to the sterilized titanium plate in a sterile culture cup. After incubation at 37 °C for 6 h, the plate was slowly removed from the culture cup and rinsed with phosphate-buffered saline (PBS, 5 mL) to remove any planktonic bacteria present on the surface. Subsequently, the titanium plate was transferred to a new sterile culture cup, and sterile saline (5 mL) was added to immerse the titanium plates. The immersed wire was placed in an ultrasonic shaker and then diluted 10,000 times. An aliquot (100 μL) of the solution was then spread over a nutrient agar medium plate (Qingdao Hope Bio, China) and cultured at 37 °C for 24 h prior to counting the colonies.

### Iodine sustained-release test

The iodine-doped titanium plates were maintained in a physiological saline solution at 37 °C, with the iodine content being assessed after 2 weeks and 1 year.

### Statistical analysis

Statistical analysis was performed using SPSS23 software (IBM, NY, USA). Analysis of variance (ANOVA) was conducted for each group after counting the colony-forming units (CFUs), and the least significant difference (LSD) test was used for pairwise comparisons. The significance level was set at 0.05, with *P* < 0.05 being considered statistically significant.

## Results

### Structural characterization of the TiO_2_ nanotube arrays on the Ti plates

**Fig. 2:**
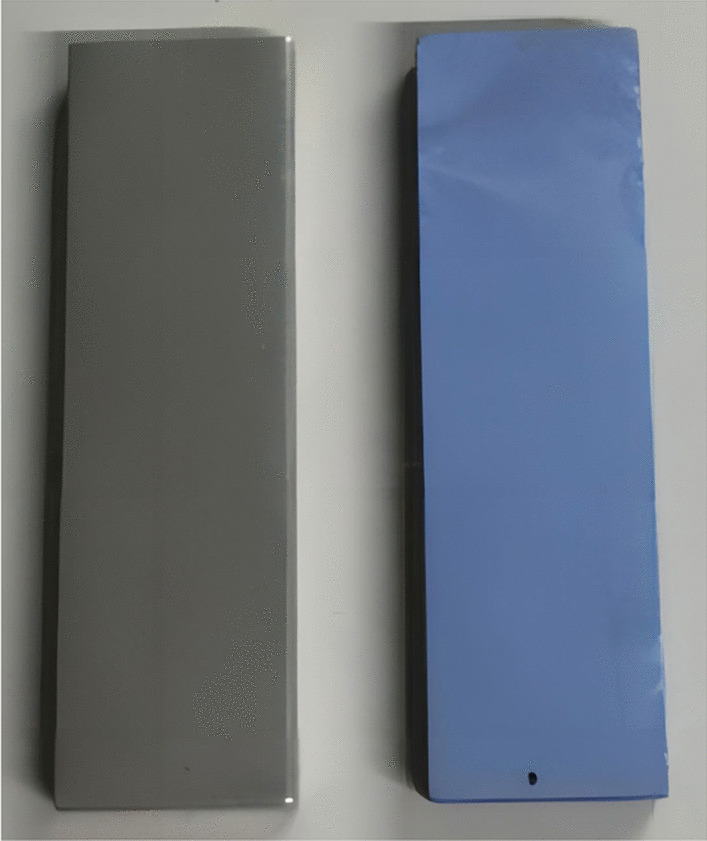
Visual characteristics of the titanium plate. The titanium plate after grinding and pretreatment (left) and the titanium plate bearing TiO_2_ nanotubes after anodization (right)

As shown in the left-hand image of Fig. 2, the oxide layer on the surface of the polished titanium Kirschner wire was smooth, without any evidence of any scratches. In addition, the TiO_2_-coated titanium plate prepared by anodic oxidation exhibited a light blue appearance along with a smooth surface (Fig. 2, right-hand image).

**Fig. 3:**
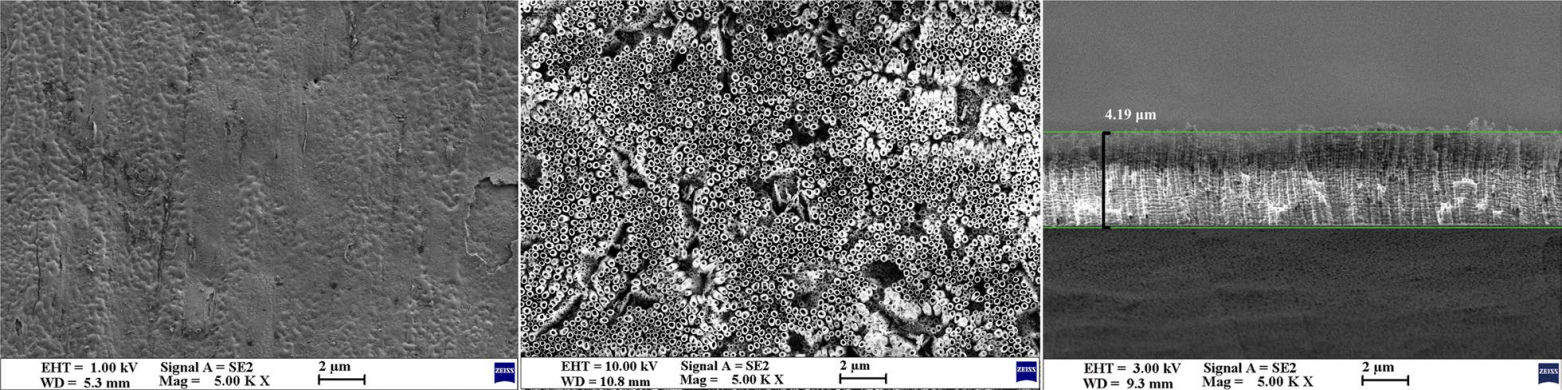
SEM images of TiO_2_ nanotube arrays. SEM observations of the growth of TiO_2_ nanotube arrays on the titanium plate surface after anodization for 10 h. Left: SEM surface view structure of titanium plate surface without anodization (×5000 magnification). Middle: SEM surface view structure of the TiO_2_ nanotube array on the titanium plate surface (×5000 magnification). Right: SEM image showing the longitudinal structure of the TiO_2_ nanotube array on the titanium plate surface (×5000 magnification)

The characteristics of TiO_2_ nanotube growth were evaluated on the surface of the titanium plate using SEM. Under a controlled voltage of 70 V in the presence of 0.50 wt.% NH_4_F in the glycerol system, the nanotubes exhibited outward growth and gradual thickening until reaching maturity and stabilizing after 10 h. As shown in Fig. 3, following anodization for 10 h, a 4.19-μm-thick layer of nanotubes developed on the surface, with diameters ranging from 210 to 240 nm.

The changes in the TiO_2_ nanotube array arrangement on the titanium surface were subsequently investigated upon adjusting the working voltage employed during the anodization method. Using a working voltage of approximately 40 V, nanotube growth was uneven, and nanotubes with diameters of ~ 140 nm were obtained. Upon increasing the working voltage to 70 V, the nanotubes grew uniformly, and their diameters were increased to 240 nm. Although the nanotube diameter increased further to 310 nm at 90 V, their distribution began to appear uneven, and partial collapse was observed by SEM imaging.

The influence of the NH_4_F concentration on the TiO_2_ nanotube array was also examined. Using a concentration of 0.30 wt.% NH_4_F glycerol, the nanotube structures failed to form. However, upon increasing the concentration to 0.50 wt.%, an extremely uniform distribution of nanotubes (~ 230 nm diameter) was observed. Upon further increase in the concentration to 0.8 wt.%, the uniformity of the nanotube distribution decreased, and the diameter also reduced to ~ 200 nm.

### Structural characterization of the iodine-doped TiO_2_ nanotube arrays

**Fig. 4:**
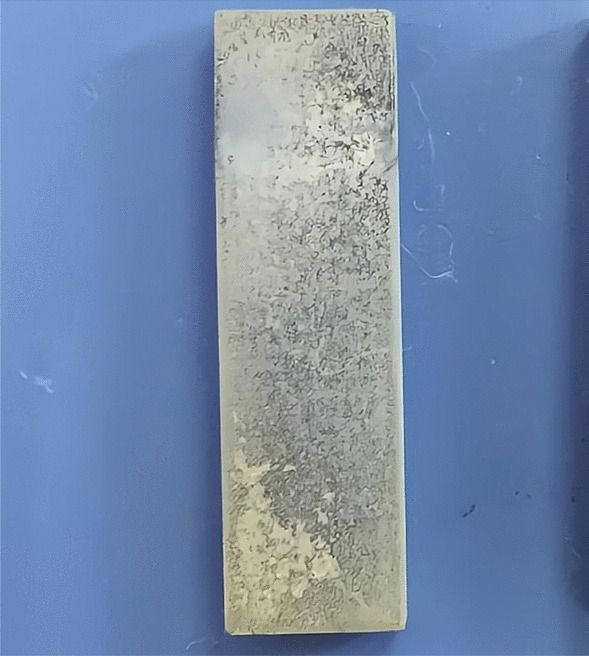
Visual characteristics of iodine-doped TiO_2_ nanotube-coated titanium plate. Appearance of the iodine-doped TiO_2_ nanotube-coated titanium plate. After the electrophoretic deposition of iodine onto the TiO_2_ nanotube arrays, a light-yellow granular coating was observed on the surface of the titanium plate

**Fig. 5:**
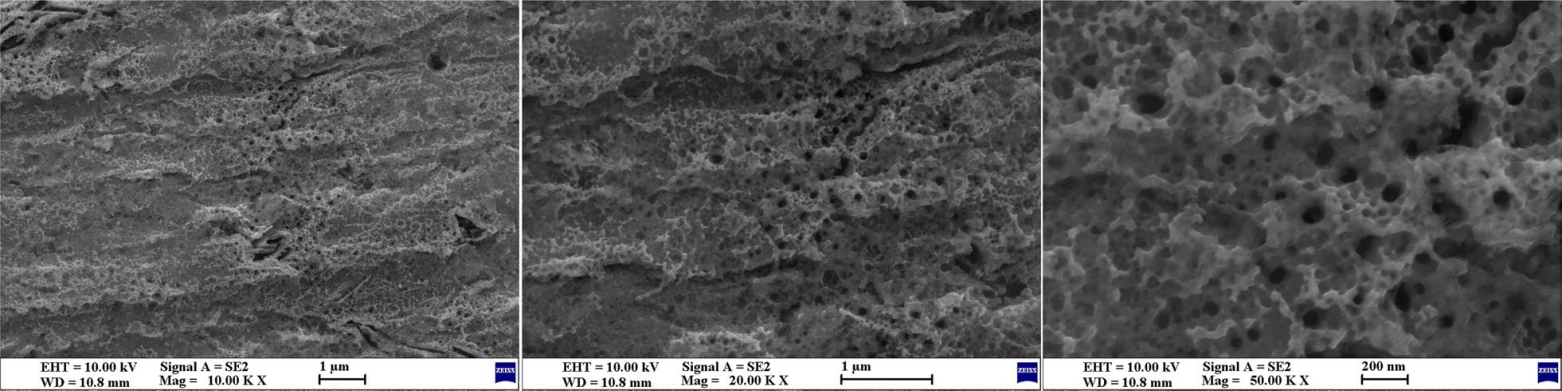
SEM images of the iodine-doped TiO_2_ nanotube structure. Microscopic representation of the iodine-doped TiO_2_ nanotube structure. At 10,000-fold (left), 20,000-fold (middle), and 50,000-fold (right) magnifications, the microscopic representation of the iodine-doped TiO_2_ nanotube structure as observed by SEM imaging. Some crystal structures were deposited on the surfaces of the nanotubes, which were tightly bound and distributed along the orifices and circumferences of the tubes

**Fig. 6:**
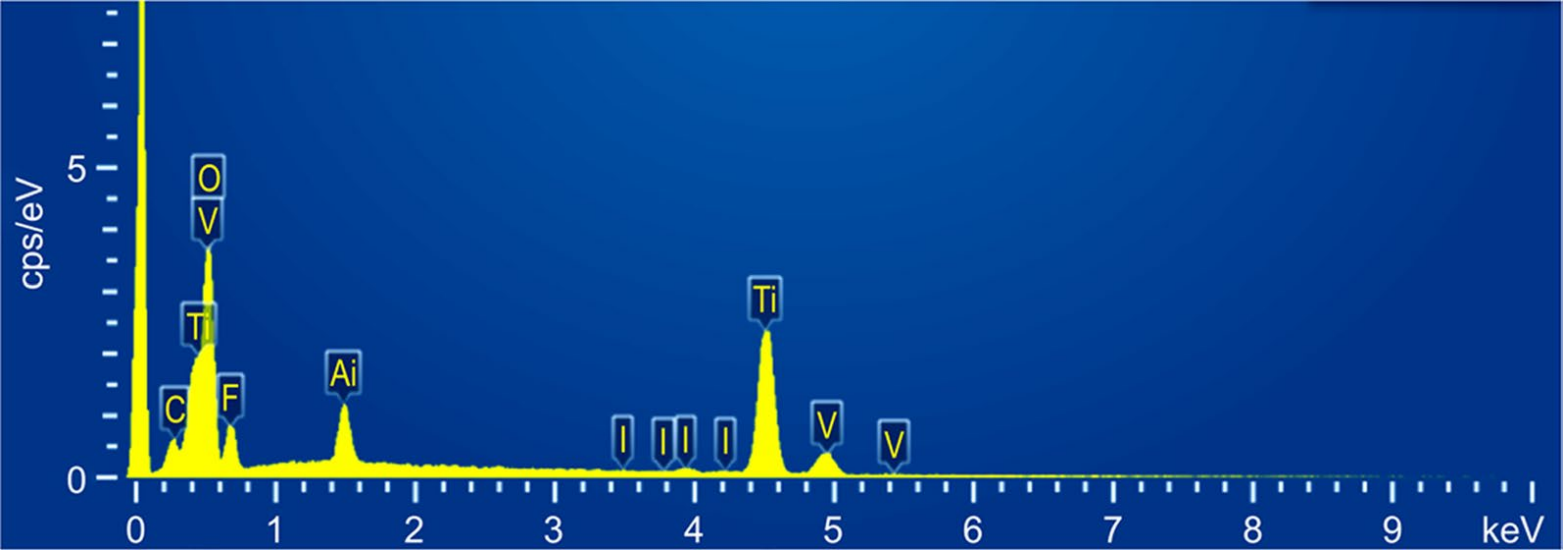
Element content analysis. Elemental content map of the titanium plate surface as determined using XRF spectrometry. The elements on the surface of the titanium plate include titanium, oxygen, fluorine, iodine, carbon, aluminum, and vanadium

**Fig. 7:**
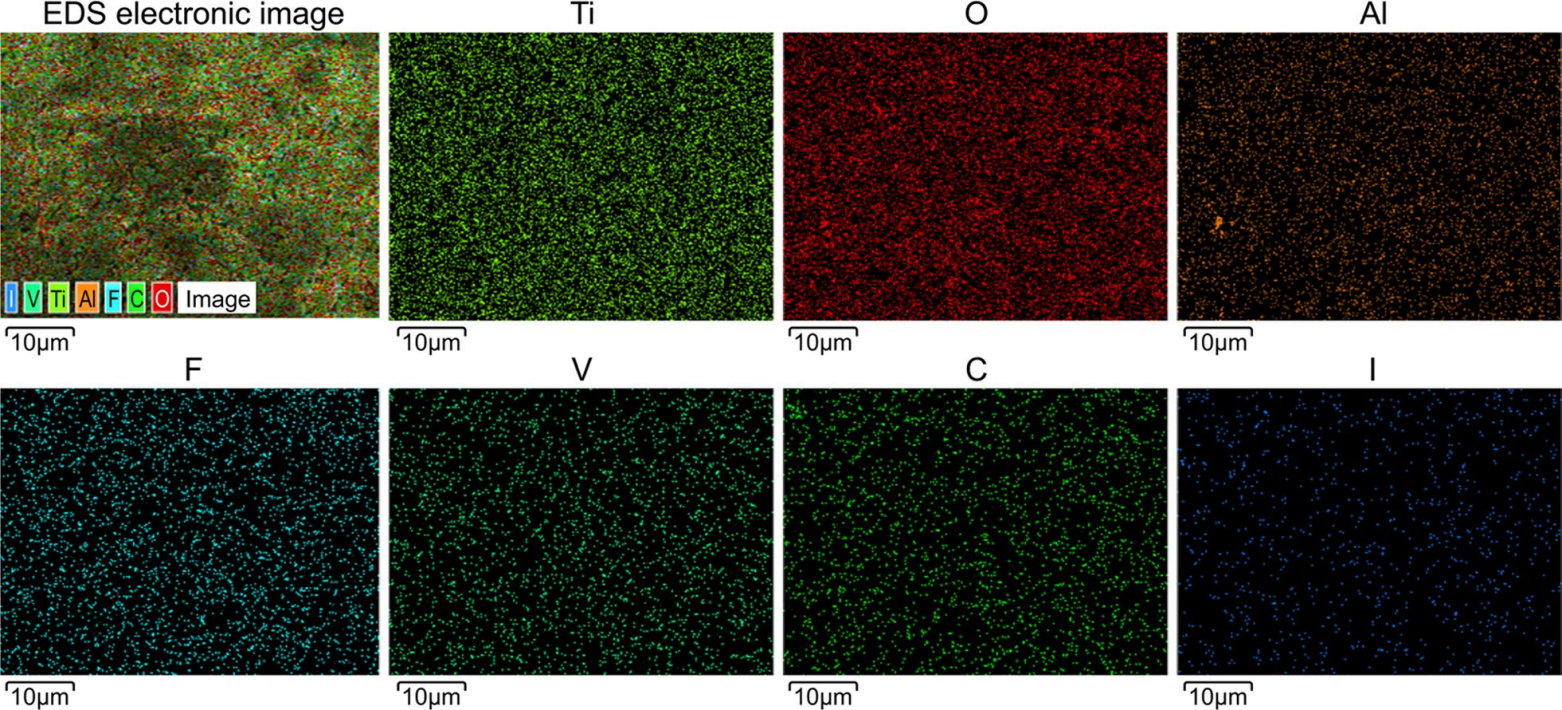
Element distribution analysis. Elemental electronic image of the titanium plate surface as determined using EDS. The elemental composition of the titanium plate surface follows a descending order: titanium, oxygen, fluorine, iodine, carbon, aluminum, and vanadium. Iodine exhibits a uniform distribution across the surface

After the electrophoretic deposition of iodine onto the TiO_2_ nanotube arrays, a light-yellow granular coating was observed on the surface of the titanium plate (Fig. 4). This coating was roughly uniform with a stable structure, which was not affected by ultrasonic cleaning. In addition, some crystal structures were deposited on the surfaces of the nanotubes, which were tightly bound and distributed along the orifices and circumferences of the tubes (Fig. 5). X-ray fluorescence spectrometry (XRF) and energy-dispersive spectrometer (EDS) confirmed the successful doping of iodine, wherein the iodine content on the surface of the titanium plate was 3.44 wt.%. As outlined in Figs. 6, 7, and Table 1, the most abundant element was titanium (51.42 wt.%), followed by oxygen (29.93 wt.%), fluorine (7.14 wt.%), and iodine (3.44 wt.%). Iodine exhibits a uniform distribution across the surface.

**Table 1:** Elemental composition of the titanium plate surface

Element	Mass fraction (wt.%)
C	3.20
O	29.93
F	7.14
Al	3.02
Ti	51.42
V	1.85
I	3.44
Total	100.00

### Antibacterial tests

**Fig. 8:**
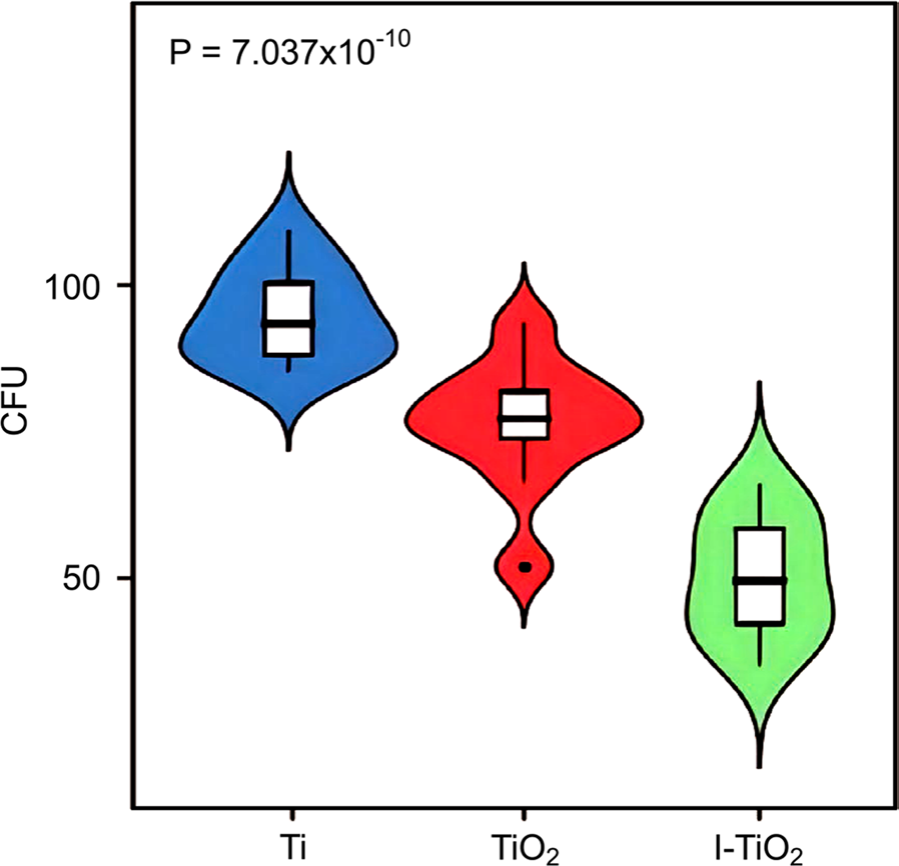
Statistical analysis. Comparison of the bacterial counts in the three experimental groups. Upon carrying out statistical ANOVA, the F value was determined to be 50.8139, and the difference was statistically significant (*P* = 7.037 × 10^−10^, < 0.05). Compared with the Ti group, the bacterial counts of the TiO_2_ and I-TiO_2_ groups decreased to varying degrees, and the differences were statistically significant (*P* < 0.05). Moreover, compared with the TiO_2_ group, the bacterial count of the I-TiO_2_ group was significantly lower (*P* < 0.05)

The bacterial counts obtained for the nutrient agar medium plate of the control group (Ti group), the TiO_2_ group, and the I-TiO_2_ group were 95.30 ± 8.07, 76.50 ± 11.36, and 50.00 ± 10.50, respectively. In addition, all *P* values were > 0.1, and the samples conformed to a normal distribution. Upon carrying out statistical ANOVA, the *F* value was determined to be 50.81, and the difference was statistically significant (*P* = 7.04 × 10^−10^, < 0.05), as shown in Fig. 8. Compared with the control group, the bacterial counts of the TiO_2_ and I-TiO_2_ groups decreased to varying degrees, and the differences were statistically significant (*P* < 0.05). Moreover, compared with the TiO_2_ group, the bacterial count of the I-TiO_2_ group was significantly lower (*P* < 0.05).

### Iodine sustained-release test

The gradual decrease in surface iodine content of the iodine-doped titanium plate was observed when stored in a saline environment at 37 ℃. After two weeks, the iodine element on the titanium plate decreased by approximately 20%, and this reduction escalated to 80% within a year.

## Discussion

Titanium alloys are known to exhibit good biological characteristics and unique physical properties that meet the necessary clinical requirements. However, because titanium is a foreign substance and an inert metal, surface adhesion and aggregation of bacteria can occur, leading to refractory periprosthetic infection [18, 19]. At present, the surface modification and coating of titanium implants are the most used methods to impart antibacterial properties to such structures [12]. Bacterial adhesion is influenced by many factors such as physical and chemical characteristics, nanomorphology, wetness, and roughness of the material surface. For instance, Kunrath et al. found that the high roughness and hydrophilicity of the implant surface increased the bacterial adhesion of the surface [20, 21]. Meanwhile, our study lacks the determination of surface roughness and wettability of the iodine-doped titanium plates. Adjustments to surface wettability, roughness, and other characteristics may alter the antibacterial performance of the iodine-doped titanium plates, necessitating further experiments to confirm these findings. As a potential coating material, TiO_2_ nanotubes are known to generate stable film layers with orderly arrangements, porous structural arrays, and high surface areas. Previously, anodic oxidation has been widely used to prepare these nanotubes because of its simple and low cost process, in addition to its ability to generate highly ordered arrays and take part in strong bonding with the titanium matrix [22]. To date, many studies have shown that the use of different nanotube morphologies can induce osteogenic differentiation and bone mineralization of bone marrow mesenchymal stem cells, wherein the ability to induce osteogenesis varies according to the nanotube diameter [23]. In addition, the inhibitory effect of TiO_2_ nanotubes on bacterial adhesion occurs mainly through the generation of reactive oxygen species, which kill bacteria; however, a photocatalytic mechanism has also been reported [24, 25]. With these considerations in mind, anodization was used in the present study to prepare TiO_2_ nanotube arrays on the surface of a titanium plate. By continuously adjusting the voltage parameters, anodization time, and concentration of the NH_4_F glycerol system, an optimal voltage of 70 V was determined for application in a 0.5 wt.% NH_4_F in glycerol system over 10 h. Under these conditions, the obtained TiO_2_ nanotube array contained structures with diameters of 210–240 nm and heights of 4.2 μm, which adopted a stable and ordered arrangement. Using SEM for morphology monitoring at different time periods, four stages of nanotube growth were identified, namely appearance, aggregation, growth, and maturation. By in vitro experiments, this modified structure exhibited a superior antibacterial activity compared to the unmodified titanium plate. Moreover, the nanotube thickness is responsible for determining the color of the nanotube coating [26], which in this case was blue.

Titanium nanocoatings prepared via the anodization oxidation approach are durable coatings that successfully block the titanium plate surface and inhibit bacterial adhesion. However, their antibacterial characteristics tend not to meet most clinical requirements. Thus, considering the drug-loading characteristics of nanotubes, the development of a safe and low-toxicity drug-loading structure was considered using these structures. For this purpose, the PVP-I complex was selected, wherein the bactericidal component is iodine. The bactericidal mechanism of iodine involves inhibition of the bacterial cell processes, the oxidation of nucleotides/amino acids and fats in the bacterial cell membrane, inhibition of the cytoplasmic enzymes of the respiratory chain, denaturation, and inactivation [27]. In vitro experiments have shown that iodine not only exhibits a wide range of antibacterial effects, but that it also antagonizes both pathogens and host inflammatory responses [28]. PVP-I has been used for decades for infection prevention [14], and it is one of the few agents that has been shown to be effective against bacteria, fungi, viruses, spores, and amoebic cysticercus in topical antibacterial experiments. Indeed, it has been demonstrated to kill a range of bacteria that are responsible for nosocomial infections, including methicillin-resistant *Staphylococcus aureus* (MRSA) [29, 30]. Thus, considering these characteristics, 3.44 wt.% iodine was doped onto the surfaces of the TiO_2_ nanotubes via the electrophoretic deposition method, as confirmed by elemental mapping and quantification. During the subsequent in vitro experiments, the antibacterial activity of the iodine-doped TiO_2_ nanotube-coated Ti plate was superior to that of the pure TiO_2_ nanotube-coated Ti plate. Previously, our group investigated the direct doping of iodine onto the surface of a titanium plate via the electrophoretic deposition method, wherein the iodine doping reached 14.48 wt.%. However, the antibacterial properties of the resulting material did not reflect this higher doping, indicating that the nanotubes also imparted a beneficial effect. Thus, subsequent experiments were carried out to increase the iodine content of the coating surface by increasing the PVP-I electrolyte concentration, altering the voltage, and prolonging the treatment time; however, the results were unsatisfactory. More specifically, altering the voltage and prolonging the electrophoresis time led to an increase in the nanotube thickness, an increase in the resistance, and a decrease in the working current, eventually leading to loss of the film structure and a reduced iodine content. Furthermore, the antibacterial experiment within this study was exclusively focused on Staphylococcus aureus for a duration of 24 h in vitro. Further investigation is required to ascertain whether altering bacterial strains and extending the antibacterial experiment time would have an impact on the antibacterial performance.

Importantly, the prepared iodine-doped TiO_2_ nanotube-coated structure is a combination of two modification modes on the surface of the titanium plate. Although antibacterial experiments have only been carried out in vitro thus far, it would be of interest to carry out in vivo experiments to determine whether the doping of iodine increases the toxic effects. For example, the release of iodine into the body could cause metabolic abnormalities related to the thyroid function. Previously, the V97 cell colony aggregation method was used to study the cytotoxicity of iodine-coated implants in vitro [31], and it was reported that, compared to stainless steel and titanium alloys, there was no specific difference in the number of cells growing on the culture dish of the iodine-coated implant, thereby suggesting that the toxic effect of iodine is low. Other studies have found that iodine does not cause changes in thyroid function in vivo, and a good safety profile was recorded in more than 20 patients [32, 33]. Moreover, the release cycle of iodine is a matter of concern. The iodine content was found to have decreased by 20% after two weeks, and by > 80% after one year. Since the incidence of implant-associated infection is concentrated in the period between surgery and wound healing, a period of two weeks can be considered a key marker. Thus, maintaining a high concentration of iodine on the surface of the titanium plate over this period should help prevent and treat acute infection after prosthesis implantation. Therefore, the iodine-loaded TiO_2_ nanotube-coated titanium plates should exhibit good safety prospects. Finally, there are still some shortcomings to the current study. For example, further experimentation is required to characterize the implant structure, including its surface roughness and wetness, and future studies should be focused on images related to the bacterial biofilm development, cellular and in vivo models, and biocompatibility evaluations.

## Conclusions

In this study, a titanium dioxide nanotube coating was prepared on the surface of a titanium plate by anodic oxidation, and iodine doping was realized by electrophoretic deposition. The iodine-doped titanium dioxide nanotube-coated titanium plate, which was produced by combining the two surface modification methods, exhibited a stable film structure and good antibacterial properties against *Staphylococcus aureus* based on the 24-h in vitro antimicrobial activity test, providing a new theoretical basis for the research and development of anti-infection implants in orthopedics.

## Data Availability

The datasets used and analysis relevant to this study are available from the corresponding author upon reasonable request.

## Abbreviations

BBF: Bacterial biofilm
PVP-I: Polyvinyl pyrrolidone-iodine
SEM: Scanning electron microscopy
PBS: Phosphate-buffered saline
ANOVA: Analysis of variance
CFU: Colony-forming unit
LSD: Least significant difference
MRSA: Methicillin-resistant *Staphylococcus aureus*
